# Surpoids et obésité dans la population au-dessus de 20 ans en milieu urbain bamakois (Mali)

**DOI:** 10.11604/pamj.2014.19.352.4381

**Published:** 2014-12-04

**Authors:** Hamidou Oumar Bâ, Ichaka Menta, Youssouf Camara, Ibrahima Sangaré, Noumou Sidibé, Seydou Doumbia, Mamadou Bocary Diarra

**Affiliations:** 1 Centre Hospitalier Universitaire Gabriel Touré, Mali; 2 Centre Hospitalier Universitaire Kati, Mali; 3 DER de Santé Publique, Faculté de Médecine et d'Odontostomatologie, Mali; 4 Centre Hospitalier «Mère-Enfant « Le Luxembourg », Mali

**Keywords:** Surpoids, obésité, adulte, population urbaine, Bamako, overweight, obesity, adult, urban population, Bamako

## Abstract

**Introduction:**

Il est question dans notre travail d’étudier le SP et l'OB et les facteurs associés dans la population âgée de 20 ans ou plus.

**Méthodes:**

Notre échantillon a été obtenu à partir d'une enquête sur les pathologies cardiovasculaires dans le District de Bamako et impliquant 2199 sujets de 5-104 ans, en sélectionnant tous les sujets âgés d'au moins 20 ans (1162). Pour chaque sujet, l'IMC, rapport taille / hanche et le tour de taille ont été déterminées. Les données ont été analysées avec SPSS 12.

**Résultats:**

L’âge moyen était de 36,86 années, 61,4% étaient des femmes, 49,7% dans le secteur informel et 38,0% avaient réalisé l'enseignement primaire. Facteurs de risque cardiovasculaires étaient l'inactivité physique (72,4%), le tabagisme (12,2%) et hypertension (26,7%). La prévalence de l'obésité était de 8,8 et 14,7% respectivement sur la base de l'indice de masse et le tour de taille.

**Conclusion:**

Le SP et l'OB sont à prendre en compte dans les mesures de politique sanitaire que dans la pratique quotidienne des professionnels de santé, il est peut-être plus utile d'utiliser plusieurs paramètres pour être à même de bien stratifier nos patients par rapport à leur risque.

## Introduction

Surpoids (SP) et obésité (OB) sont liés à un accroissement du risque cardiovasculaire et le bénéfice potentiel de leur réduction a été souligné [1–4]. La mesure la plus communément utilisée est l'indice de masse corporel (IMC) à côté du tour de taille (TT) et du rapport tour de taille/tour de hanche (TT/TH). La prévalence du SP et l'OB augmente aussi bien dans les pays développés que les pays en développement et de nombreux facteurs sont incriminés face à cette situation qualifiée d’épidémie et les données diffèrent selon l′outil de mesure utilisé [5–10]. Selon les estimations de l'organisation mondiale de la santé (OMS) en 2008, plus de 300 millions de femmes et près de 200 millions d'hommes étaient obèses. L'OMS prévoit que d'ici 2015, quelque 2,3 milliards d'adultes auront un surpoids et plus de 700 millions seront obèses [1]. Au Mali les données disponibles sont surtout hospitalières issues de thèse de Médecine avec des prévalences de 23,4% parmi les patients souffrant de cardiopathies ischémiques, les prévalences du SP et de l′OB basées sur l′IMC estimées resp. à 11,9 et 15,3%, ce qui est moins bien corrélé au risque cardiovasculaire que les prévalences basées sur le tour de taille. La nécessité d′avoir des données de référence basées sur la population générale a motivé la réalisation de cette étude du SP et de l′OB et de leurs facteurs associés en utilisant 2 outils de mesure que sont l′IMC et le TT.

## Méthodes

### Description de l′échantillon

L′étude a été réalisée dans les six communes du District de Bamako, capitale du Mali. Bamako est une ville cosmopolite avec toutes les ethnies du pays représentées et aussi des populations de pays voisins. Comme beaucoup de capitales en Afrique Sub-Saharienne, il est observé un changement de mode de vie avec une sédentarité accrue, une alimentation augmentant le risque cardiovasculaire.

### Collecte des données

L′échantillon est issu de l′enquête sur les pathologies cardiovasculaires dans le District de Bamako, réalisée de Novembre à Décembre 2002. Pour rappel l′enquête avait été conçue selon l′approche STEP de l′OMS [11] et réalisée selon la stratégie porte à porte en partant de la concession du chef de quartier. Tous les sujets âgés de 20 ans ou plus ont été inclus dans notre étude, soit 1162 sujets. En plus de l′interrogatoire, le poids, la taille, le tour de taille et de hanche, de même que la pression artérielle étaient obtenus selon les règles.

### Analyse des données

L′OB basée sur l′IMC a été appelée OB générale (OBG) [12] et l′OB centrale (OBC) basée sur le tour de taille qui est un meilleur paramètre du risque cardiovasculaire [12–14]. SP et OB générale ont été définis par l′IMC resp. supérieur ≥ 25 Kg/m^2^ et ≥ 30 Kg/m^2^. SP et OB centrale de même ont été définis resp. par un TT ≥ 80 cm et 88 cm pour les femmes et ≥ 90 cm et ≥ 102 cm pour les hommes. Les données collectées ont été saisies sur Epi Info 6.04d et vérifiées quant à leur validité sur 10% des formulaires. L′analyse statistique a été réalisée avec le logiciel SPSS 12, les résultats exprimés en moyenne et déviation standard. Les tests utilisés le t-test pour les variables continues et le Chi-2 ou le Fisher-Test pour les variables discontinues. Une régression logistique a été utilisée pour rechercher les meilleurs prédicteurs du SP et de l′OB.

## Résultats

### Description de l′échantillon

La moyenne d′âge était de 36,86 ans (20-104) et 61,4% de l′échantillon était de sexe féminin. La tranche d′âge au-dessous de 30 ans représentait 43,8%, 50,1% des sujets enquêtés exerçaient dans le secteur informel et 58,6% avaient un niveau d′instruction primaire (Tableau 1). Les facteurs de risque cardiovasculaires retrouvés étaient la sédentarité (72,4%), le tabagisme (12,2%) et l′hypertension artérielle (HTA) 26,8%.

**Tableau 1:** Caractéristiques de l’échantillon

Variables		Sexe		N (%)
		Masculin	Féminin	
Tranche d’âge (ans)	< 30	42,5	44,6	509 (43,8)
	30-44	32,1	31,7	370 (31,8)
	45-59	11,6	10,8	129 (11,1)
	>= 60	13,8	12,9	154 (13,3)
Profession	Travailleur de bureau	31,1	12,8	234 (20,3)
	Ouvrier	35,7	7,0	209 (18,8)
	Etudiant	15,8	8,7	132 (11,4)
	Secteur informel	16,3	71,6	578 (50,1)
Niveau d'instruction	Primaire	48,4	67,1	442 (58,6)
	Secondaire	32,3	28,6	228 (30,2)
	Supérieur	19,4	4,4	84 ( 11,1)
Sédentarité	Oui	68,7	84,6	841 (78,4)
	Non	31,3	15,4	232 (21,6)
Tabagisme	Fumeur actif	30,6	0,7	142 (12,7)
	Non fumeur	68,0	99,3	972 (86,8)
	Fumeur occasionnel	1,3	0,0	6 (0,5)
HTA	Oui	25,7	27,4	308 (26,8)
	Non	74,3	72,6	842 (73,2)

### Prévalence du SP et de l′OBG ( Tableau 2, Tableau 3, Tableau 4)

La prévalence du SP était de 16,6% et celle de l′OB 8,8% (Tableau 2). Les communes 2, 3 et 5, la tranche d′âge 45-59 ans et le sexe féminin étaient plus représentés (Tableau 3). De même les travailleurs de bureau, ceux du secteur informel, les non-fumeurs et les sujets hypertendus étaient plus touchés. Le niveau d′instruction, la sédentarité ne différaient pas significativement. En analyse multi variée le sexe et l′hypertension artérielle étaient associés au SP et l′OB: le sexe masculin était protecteur tandis que l′HTA était facteur de risque (3,7 x) (Tableau 4)

**Tableau 2:** Prévalence du surpoids et de l'Obésité

Variables		Sexe		N (%)
		Masculin	Féminin	
Obésité générale ( IMC)	Poids normal	45,7	54,3	738 (63,5)
	Surpoids	29,5	70,5	193 (16,6)
	Obésité	13,7	86,3	102 (08,8)
Obésité centrale (TT)	Poids normal	49,3	50,7	804 (69,2)
	Surpoids	23,0	77,0	187 ( 16,1)
	Obésité	05,8	94,2	171 ( 14,7)

**Tableau 3:** Répartition du Surpoids et de l'Obésité en fonction de l'IMC

		Evaluation de l'IMC			Total	P
Variables		Normal	SP	OB		
Commune	1	63,3	16,9	9,6	177	< 0,0001
	2	57,4	19,1	12,5	136	
	3	50,9	16,7	12,0	108	
	4	74,0	9,6	7,2	208	
	5	57,5	19,5	10,2	266	
	6	69,7	17,6	4,9	267	
Tranche d’âge ( ans)	< 30	74,3	11,6	4,3	509	< 0,0001
	30-44	55,9	20,3	12,2	370	
	45-59	46,5	23,3	14,7	129	
	>= 60	60,4	18,8	10,4	154	
Sexe	M	75,1	12,7	3,1	449	< 0,0001
	F	56,2	19,1	12,3	713	
Profession (1153)	Travailleur de Bureau	57,3	20,1	9,8	234	< 0,0001
	Travailleur manuel	70,3	14,4	5,7	209	
	Scolaire	83,3	6,1	3,8	132	
	Secteur informel	59,0	18,7	10,4	578	
Niveau d’étude	Primaire	59,7	18,6	10,0	442	0,105
	Secondaire	64,9	13,6	8,3	228	
	Supérieur	72,6	17,9	4,8	84	
Sédentarité	Sédentaire	62,9	16,6	9,5	841	0,269
	Non sédentaire	64,7	16,8	5,6	232	
Tabagisme	Actif	78,9	10,6	2,1	142	0,007
	Non fumeur	62,4	17,1	9,4	972	
	Occasionnel	66,7	16,7	0	6	
HTA	Hypertension	47,2	19,2	18,2	307	< 0,0001
	Normotendu	69,5	5,0	15,9	843	

**Tableau 4:** Surpoids et obésité en analyse multi variée

Mesures	Variables	Chi-2	P	OR	95% IC
IMC	Masculin vs féminin	3,832	0,05	0,350	0,123-1.001
	Hypertension	13,052	< 0,0001	3,713	1,822-7,564
TT	Masculin vs féminin	6,096	0,014	0,241	0,078-0,746

### Prévalence du SP et de l′OBC ( Tableau 2, Tableau 4, Tableau 5)

La prévalence du SP était de 14,7%, celle de l′OB 16,1% ( Tableau 2). En analyse uni variée (Tableau 5) certaines caractéristiques (commune, tranche d′âge, sexe, profession, niveau d′instruction, sédentarité, tabagisme et HTA) présentaient des différences significatives. En analyse multi variée (Tableau 4) le sexe était associé au SP et à l′OB. Le sexe masculin était protecteur.

**Tableau 5:** Répartition du surpoids et de l'obésité en fonction du tour de taille

Variables		Evaluation de l'IMC			Total	P
		Normal	SP	OB		
Commune	1	72,9	15,3	11,9	177	0,005
	2	67,6	16,2	16,2	136	
	3	64,8	21,3	13,9	108	
	4	79,8	8,2	12,0	208	
	5	61,3	19,2	19,5	266	
	6	68,9	17,6	13,5	267	
Tranche d’âge( ans)	< 30	81,9	11,8	6,3	509	< 0,0001
	30-44	59,5	18,9	21,6	370	
	45-59	55,0	22,5	22,5	129	
	>= 60	62,3	18,2	19,5	154	
Sexe	M	88,2	9,6	2,2	449	< 0,0001
	F	57,2	20,2	22,6	713	
Profession	Travailleur de Bureau	70,1	18,8	11,1	234	< 0,0001
	Travailleur manuel	81,3	12,9	5,7	209	
	Scolaire	91,7	3,0	5,3	132	
	Secteur informel	59,5	19,0	21,5	578	
Niveau d’étude	Primaire	66,1	18,6	15,4	442	0,001
	Secondaire	78,5	11,0	10,5	228	
	Supérieur	81,0	15,5	3,6	84	
Sédentarité	Sédentaire	66,6	17,5	15,9	841	< 0,0001
	Non sédentaire	79,7	12,1	8,2	232	
Tabagisme	Actif	90,1	7,0	2,8	142	< 0,0001
	Non fumeur	66,6	17,6	15,8	972	
	Occasionnel	100,0	0,0	0,0	6	
HTA	Hypertension	54,1	22,1	23,8	307	< 0,0001
	Normotendu	74,6	14,0	11,4	843	

## Discussion

**Prévalence de l′OB**: dans notre échantillon, 8,8% et 14,7% avaient respectivement une OBG et OBC. Ces prévalences sont inférieures à celles observées dans les pays développés comme les Etats Unis en 2004 avec 32,2% [2], l′Allemagne avec 23,9% [15], 16,9% et 46,9% en France [16]. En Afrique et particulièrement dans la sous-région ouest-africaine la prévalence de l′OB est estimée à 10,5% [17], jusqu′à 32% pour l′OBC [7]. Comme dans d′autres études [17–19], l′OB est plus fréquente dans le sexe féminin. Dans notre échantillon l′OBC était plus importante que l′OBG (14,7% contre 8,8%), résultat comparable à celui d′autres études [7, 9, 14, 17]. Ceci est particulièrement important car l′OBC est mieux corrélée au risque cardiovasculaire que l′IMC [12]. Ces données suggèrent que malgré des prévalences relativement faibles, nos sujets n′en sont pas moins à risque cardiovasculaire faible.

**Etude de l′OBG**: contrairement aux données suggérées en analyse uni variée, seuls le sexe et l′HTA étaient significativement associés à l′OBG. Ainsi le sexe masculin était protecteur ( OR 0,350) tandis que l′HTA représentait un grand risque ( OR 3,7)

**Etude de l′OBC**: l'OBC était plus retrouvée que l′OBG comme dans d′autres études [5, 7, 15]. Nos prévalences sont plus faibles que celles de pays voisins [5, 7, 11, 19], ceci pouvant s′expliquer par les différences dans le régime alimentaire. En analyse multi variée seul le sexe masculin était protecteur (CR 0,241) et confirme la prévalence de l′OB dans la population féminine et cela en accord avec la majorité des études. Une explication pourrait être la durée de l′HTA avec observation d′un régime avec perte de poids grâce au régime. Mais nous n′avons pas pu vérifier cela dans cette étude.

**Limites de l′étude**: l’étude a souffert de l′absence des données biologiques pour mieux apprécier le risque cardiovasculaire des sujets âgés ayant participé à l′enquête. La raison principale était d′ordre financier.

## Conclusion

Le surpoids et l'obésité sont à prendre en compte dans les mesures de politique sanitaire que dans la pratique quotidienne des professionnels de santé, qui chacun à des degrés différents aura à suivre des patients en surpoids ou obèses. La façon de mesurer cet état donnant des résultats différents, il est peut être plus utile d'utiliser plusieurs paramètres pour être à même de bien stratifier nos patients par rapport à leur risque.
